# 535. Comparison of Outcomes in Patients Positive for Severe Acute Respiratory Syndrome Coronavirus 2 (SARS-CoV-2) Infection After Monoclonal Antibody Therapy (MAT) with Bamlamivimab or Casirivimab-Imdevimab

**DOI:** 10.1093/ofid/ofab466.734

**Published:** 2021-12-04

**Authors:** Courtney Nichols, Mark Lustberg, Mohammad Mahdee Sobhanie, Joy Lehman, Erica E Reed, Nicholas E Kman, Mark Conroy, Michael Dick, James N Allen, Jonathan Parsons, Carlos Malvestutto

**Affiliations:** 1 OSU Wexner Medical Center, Columbus, Ohio; 2 The Ohio State University, Columbus, Ohio; 3 The Ohio State University Wexner Medical Center, Columbus, OH; 4 Ohio State University Wexner Medical Center, Columbus, Ohio; 5 The Ohio State University College of Medicine, Columbus, Ohio; 6 Ohio State University, Columbus, Ohio

## Abstract

**Background:**

Limited options currently exist for treatment of patients diagnosed with symptomatic coronavirus 2019 (COVID-19). Monoclonal antibody therapy (MAT) has been investigated as a therapeutic option for symptomatic COVID-19 patients in the outpatient setting at high-risk for progression to severe disease based on emergency use authorization (EUA) criteria. No published studies have compared outcomes for patients treated with different MAT for COVID-19.

**Methods:**

This was a single-center, retrospective cohort study at The Ohio State University Wexner Medical Center to compare COVID-19-related emergency room (ER) visits, admissions, and mortality at 30 days after MAT infusion for adult patients with symptomatic SARS-CoV-2 between November 16, 2020 and February 2, 2021 who received bamlanivimab versus those who received casirivimab-imdevimab. Statistical analysis used logistic regression analysis to determine the odds ratio (OR) to evaluate the relationship between patient characteristics, MAT, and outcomes.

**Results:**

The cohort included 943 patients with SARS-CoV-2 who received MAT, including 658 patients who received bamlanivimab and 285 who received casirivimab-imdevimab. Outcome results between patients who received bamlanivimab and casirivimab-imdevimab showed no statistically significant difference seen in the number of COVID-19 related ER visits (3.2% vs 3.5%, p = 0.80), hospital admissions (4.6% vs 2.8%, p = 0.21), or mortality (0.5% vs 0.7%, p = 0.63). Multivariate analysis showed no statistically significant difference in outcomes between the groups when accounting for potential confounders. As reflected in the Table, chronic lymphocytic leukemia (CLL), gender, and asthma were associated with increased COVID-19 related ER visit within 30 days of infusion and age, chronic obstructive pulmonary disease, CLL, and lupus were associated with increased risk for COVID-19 related admission within 30 days of infusion. Age and obesity with body mass index greater than 35 mg/kg^2^ were associated with increased risk for COVID-19 related mortality at 30 days.

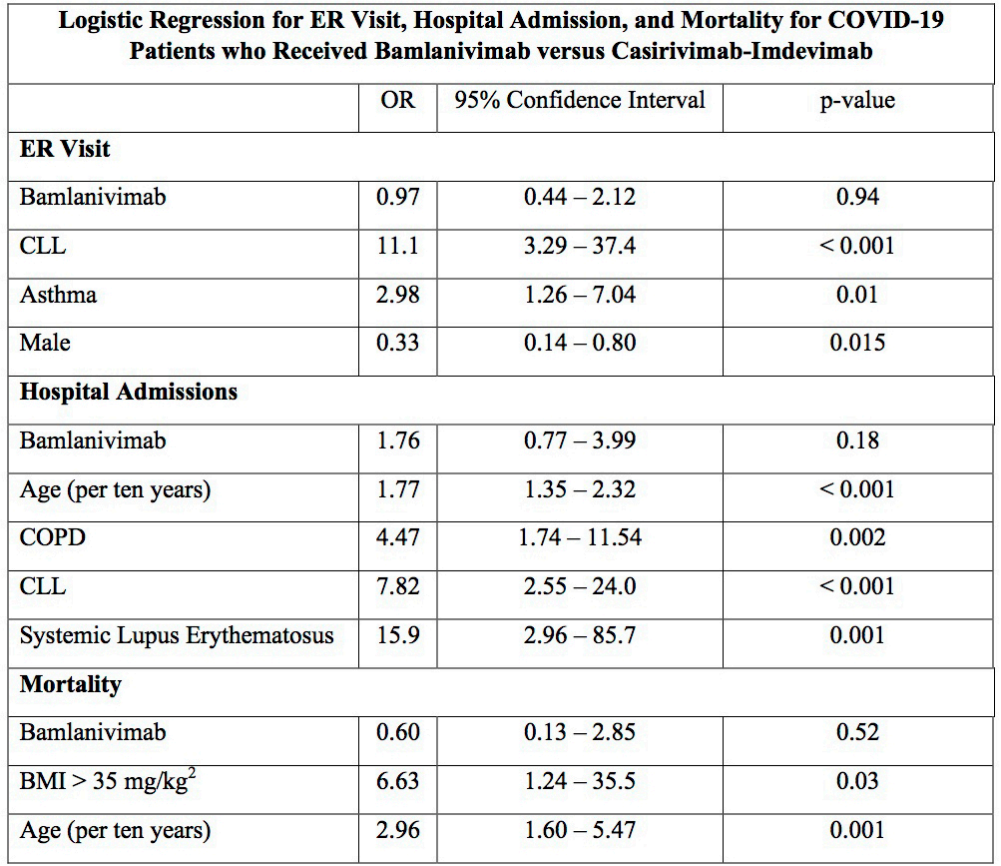

**Conclusion:**

COVID-19 related outcomes were similar when comparing patients with COVID-19 treated with bamlanivimab versus those treated with casirivimab-imdevimab.

**Disclosures:**

**Mohammad Mahdee Sobhanie, M.D.**, **Regeneron** (Scientific Research Study Investigator)**Regeneron** (Scientific Research Study Investigator, Was a sub-investigator for Regeneron 2066 and 2069) **Carlos Malvestutto, M.D.**, **Lilly** (Scientific Research Study Investigator)**Regeneron Inc.** (Scientific Research Study Investigator)**ViiV Healthcare** (Advisor or Review Panel member)

